# Regulation of Plant Mineral Nutrition: Transport, Sensing and Signaling

**DOI:** 10.3390/ijms161226198

**Published:** 2015-12-11

**Authors:** Hatem Rouached, Lam-Son Phan Tran

**Affiliations:** 1 Biochimie et Physiologie Moléculaire des Plantes Research Unit, Montpellier SupAgro, 2, Place Pierre Viala 34060 Montpellier Cedex 2, France; 2 Signaling Pathway Research Unit, RIKEN Center for Sustainable Resource Science, 1-7-22 Suehiro-cho, Tsurumi-ku, Yokohama, 230-0045, Japan

Limitation in crop yield productivity significantly contributes to the pressing problem of food security and malnutrition worldwide. The productivity of higher plants depends on the availability of various minerals in the soil, known as macro- and micro-nutrients. The green revolution that started in the 1960s has been associated with excessive use of fertilizers, a costly practice neither eco-friendly, nor sustainable. For these reasons, there has been long-standing interest in trying to find ways of maximizing crop yield with reduced fertilizer input. To achieve this objective, we first need to develop a better understanding on how plants regulate the homeostasis of different nutrients and their crosstalk. The research emphasis so far has been on elucidating the molecular physiology of individual nutritive elements. It appears that plants have developed highly effective transport, sensing and signaling systems to ensure acquisition of minerals, and have evolved various adaptive strategies to cope with toxic heavy metals. This Special Issue of the *International Journal of Molecular Sciences* (*IJMS*) specifically addresses an important component of the regulation of plant mineral nutrition: transport, sensing and signaling. The Special Issue presents a series of studies (four research papers) and reviews (three), covering different aspects of the homeostasis of essential (e.g., carbon, C; nitrogen, N; iron, Fe; zinc, Zn; and magnesium, Mg) and non-essential ions (e.g., cadmium, Cd and lead, Pb).

Magnesium (Mg) is a metallic element, which is vital for human and plant life. Mg is absorbed through plant’s roots from soil. There is often not sufficient bioavailable Mg in soil, and thus it is necessary to use fertilizer in order to supply additional Mg to plants. A significant progress in cloning genes involved in Mg transport from soil to plants and in regulation of plant responses to Mg deficiency has been achieved. In the current Special Issue, Kobayashi and Tanoi (2015) [1] have reviewed the current status of research on Mg homeostasis and the related transporters. Recent progress in physiological characterization of the plant *MRS2* gene family as well as fundamental investigations of the molecular mechanism of the action of bacterial CorA proteins were described in their review.

Other metal elements that are required for plant growth are the zinc (Zn) and iron (Fe). Deficiency in both elements affects up to 2 billion people worldwide according to the World Health Organization’s statistics, with Fe deficiency alone causing approximately 800,000 child deaths per year [2]. The widespread occurrence of deficiency in micronutrients, such as Zn and Fe, in human population is attributable to low dietary intake. The most alarming situations are particularly encountered in regions with predominant cereal-based diets (Borrill *et al.*, 2014 [3]). Indeed, cereal grains, such as rice and wheat, are inherently very low in contents and bioavailability of Zn and Fe (Borrill *et al.*, 2014 [3]). Thus, increasing the grain-filling with Zn and Fe is of primary interest. Various approaches, including enhancement of the uptake and transport of these elements in different plants organs, particularly in grains, and increasing the contents of metal chelators, such as nicotianamine and 2′-deoxymugineic acid, have been used to achieve this goal. Nevertheless, it is important to note that a non-essential/toxic ion, namely cadmium (Cd), is also transported in plants through the same transport system as Zn and Fe. Yoneyama *et al.* (2015) [4] presented an elegant review on the mechanisms underlying Zn, Fe and Cd transport and accumulation in rice plant. This knowledge is important for designing strategies for Zn and Fe biofortification, and for biosecurity programs (e.g., reducing Cd content).

In the regulation of ion transport in plants, a very important question remains to be unanswered: can a plant distinguish different metals at the uptake or transport steps? The response seems to be affirmative for some transport steps based on an example investigated by Yamaguchi *et al.* (2015) [5], in which the authors studied two metals, namely nickel (Ni) and cobalt (Co), that have similar chemical properties and are accumulated at high concentration in *Clethra barbinervis*. By performing a series of physiological experiments, including a hydroponic split-root experiment using Ni and Co solutions, Yamaguchi *et al.* (2015) [5] suggested that *C*. *barbinervis* was able to distinguish Ni and Co during transport and accumulation in the leaves, but not during their uptake through the root system. These findings open a way to design strategies to reduce heavy metal accumulation by a better control of the transport system.

Living organisms have evolved additional mechanisms to protect themselves from or tolerate heavy metals. Cyanobacteria, the only known prokaryotes that perform oxygen-evolving photosynthesis, are often challenged by heavy metals. To acquire high amount of various metals for growth, cyanobacteria are frequently affected by drastic changes in metal availabilities. Metal sensors, transporters and storages have been characterized and appeared to also be involved in defenses against oxidative stresses, and *vice versa*. Recent data point towards the importance of the crosstalk between the response to oxidative and metal stresses for the cyanobacteria survival. This knowledge is nicely summarized in Cassier-Chauvat and Chauvat (2014) [6].

Elucidation of the genetic architectures of plant responses to heavy metal stress has become possible owing to the availability of advanced molecular technologies, such as Methylated DNA Immunoprecipitation-sequencing (MeDIP-seq). Using this method, Ding *et al.* (2014) [7] revealed the genome-scale DNA methylation patterns of maize roots in response to lead (Pb) exposure, and identified candidate genes that potentially regulate root dynamic development under Pb stress. According to their results, the average methylation density was the highest in CpG islands (CGIs), followed by the intergenic regions. Additionally, their finding indicated that within the gene body, the methylation density of the introns was higher than those of the UTRs and exons.

With the aim to increase the rice productivity through enhancement of nitrogen (N) uptake and N use efficiency, Bao *et al.* (2015) [8] studied the function of the *AMT1-3* gene encoding a high affinity NH_4_^+^ transporter using the gain-of-function approach. Unexpectedly, the authors found that rice transgenic plants overexpressing the *AMT1-3* gene showed reduced N uptake and poor plant growth in comparison with wild-type, which was explained by the carbon (C) and N metabolic imbalance in leaves of transgenic plants. The authors suggested that constitutively maintaining high expression of the *AMT1-3* gene in transgenic rice plants might make the AMT1-3 protein act as a sensor that mistakenly sends a signal of N starvation to transgenic plants, leading to poor N uptake and plant growth. A complex crosstalk between N and C is emerging in the face of new data. In this context, Bao *et al.* (2015) [9] showed the importance of the C and N balance during rice growth and demonstrated the involvement of a key enzyme, namely the glutamine synthetase 2 (GS2), in this process. Combination of various genomic approaches will help better understand the molecular basis and key players in C and N homeostases and crosstalk in plants in future.

Many research works aimed to elucidate the molecular mechanisms controlling ion (either essential or not) contents in plants have been performed using diverse plant systems. It is clear that much remains to be discovered to reach the aims of improving crop yield production, reducing the use of fertilizers and limiting the heavy metal accumulation in the staple parts of plants.
